# Retraction Note: Hypermethylation of the 5′ CpG island of the *p14*^*ARF*^ flanking exon 1β in human colorectal cancer displaying a restricted pattern of p53 overexpression concomitant with increased MDM2 expression

**DOI:** 10.1186/s13148-023-01504-x

**Published:** 2023-05-17

**Authors:** Christine Nyiraneza, Christine Sempoux, Roger Detry, Alex Kartheuser, Karin Dahan

**Affiliations:** 1 Center for Human Genetics, Université Catholique de Louvain, Cliniques universitaires Saint-Luc, Avenue Hippocrate 10, B-1200 Brussels, Belgium; 2 Department of Pathology, Université Catholique de Louvain, Cliniques universitaires Saint-Luc, Avenue Hippocrate 10, B-1200 Brussels, Belgium; 3 Colorectal Surgery, School of Medicine, Université Catholique de Louvain, Cliniques universitaires Saint-Luc, Avenue Hippocrate 10, B-1200 Brussels, Belgium; 4 Institute of Pathology and Genetics, Avenue Georges Lemaître 25, 6041 Gosselies, Belgium

## Retraction note: Clinical Epigenetics 2012, 4:9 http://www.clinicalepigeneticsjournal.com/content/4/1/9

The Editor-in-Chief has retracted this article. After publication, concerns were raised regarding similarities among different parts of the methylation PCR gel image in Fig. 1B. Specifically, the two lanes representing the second Tumor (T) sample and the positive control (CTL+) appear highly similar. In addition, the unmethylated (U) lane of the CTL+ group appears to be repeated multiple times in the negative control (H2O) group.

The authors have provided some of the raw data used to produce this image, which confirmed that the image was a composite, and have been unable to sufficiently justify the similarity between the T and CTL+ groups.

The Editor-in-Chief therefore no longer has confidence in the presented data. Christine Nyiraneza, Christine Sempoux, Alex Kartheuser and Karin Dahan do not agree to this retraction. The Publisher has not been able to obtain a current email address for Roger Detry.

